# Study of Nano h-BN Impact on Lubricating Properties of Selected Oil Mixtures

**DOI:** 10.3390/ma15062052

**Published:** 2022-03-10

**Authors:** Wiesław Urbaniak, Tomasz Majewski, Iwona Powązka, Grzegorz Śmigielski, Aneta D. Petelska

**Affiliations:** 1 Faculty of Mechatronics, Kazimierz Wielki University, Chodkiewicz 30, 85-867 Bydgoszcz, Poland; wurban@ukw.edu.pl (W.U.); gsmigielski@ukw.edu.pl (G.Ś.); 2 Faculty of Mechatronics, Armament and Aerospace, Military University of Technology, Kaliskiego 2, 01-489 Warsaw, Poland; tomasz.majewski@wat.edu.pl; 3 SILESIA OIL SP. Z O.O., Wapienna 2, 43-174 Łaziska Górne, Poland; i.powazka@silesia-oil.com.pl; 4 Faculty of Chemistry, University of Bialystok, Ciolkowskiego 1K, 15-245 Bialystok, Poland

**Keywords:** nanomaterials, hexagonal boron nitride, lubricity, friction, PAO4, CB30

## Abstract

Our experiments aimed to study the influence of layered materials with nanometric-scale particles, which are part of lubricant oils, on their tribological properties. The object of this study was a lubricant oil made using base oil PAO4, which contained nanoparticle hexagonal boron nitride (nano h-BN) and a dispersant based on succinic acid imide. Comparative tests for engine oil (CB30) were also performed. The paper presents the method of preparing the test material and the tribological test results, including wear spot diameter (wear mark), limit wear load, and seizure load. The test results obtained demonstrate that nano-hexagonal boron nitride improves the tribological properties of lubricant oils. However, oil preparation and the quantitative selection of components markedly influence the results.

## 1. Introduction

One of the main operational problems of machines is the wear of the friction surfaces of the tribological node. Numerous studies have been undertaken to reduce this harmful phenomenon. One of the main operational problems of machines is the wear of the friction surfaces of the tribological node. Numerous studies have been undertaken to reduce this harmful phenomenon. In the literature, there are attempts to develop a simple model of this phenomenon, which mainly takes into account abrasives [1,2,3], adhesive [4,5], physicochemical (hydrogen, oxidizing), fatigue, including abrasive and corrosive [5,6,7]. Excessive surface roughness may cause the surface layer to shear, accompanied by an increase in temperature, accelerating the wear process, which is due to the Bowden theory, which predicts that the friction force is influenced by, e.g., the shear force of metallic connections. Greater roughness is associated with forming a larger cross-section of the friction furrow. Shear as a plastic deformation process is accompanied by an increase in temperature [5,7].

Physicochemical processes can negatively affect the oxidation of the surface layer or the accumulation of hydrogen on it [5,6,7]. Thus, the lubricant used, which protects the friction elements against excessive wear, significantly influences the frictional conditions. The condition of their surfaces and the boundary layer of the lubricant plays a significant role in this process. A solution that would protect against negative phenomena resulting from the process of friction of cooperating elements should, among others:

- ensure the lowest possible friction;
- fill the surface discontinuities that have arisen or appeared (smoothing out roughness);
- dissipate heat well from the friction node;
- be as least chemically aggressive as possible.

**Figure 1:**
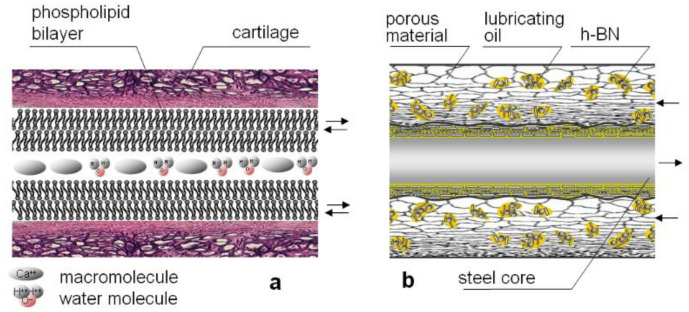
Model of lamellar lubrication in (**a**)—natural (biological) bearing and (**b**)—oil-colored porous bearings with the addition of h-BN [7].

Studies observed in nature and confirmed in literature reports show that a good solution may be using the so-called lamellar lubrication mechanism in biological joints [6,7]. Figure 1 shows a diagram of a natural (biological) bearing and a sintered porous bearing lubricated with oil with the addition of hexagonal boron nitride.

While phospholipids are responsible for very good lubricating conditions and possible repair of the biological joint, in the case of tribological nodes, such functions can be fulfilled by the so-called layered additives. There are many layer additives, often called solid lubricants [6,7].

The effects of additives improving lubricity in both oils and solid lubricants have been studied for many years. These studies indicate that adding so-called layered materials to a lubricant oil improves its tribological properties. A characteristic quality of layered materials is that parallel layers, made up of individual atoms connected by strong covalent bonds, are connected by weak Van der Waals forces. These layers can easily move relative to each other, granting the materials low friction coefficients [8,9,10]. This group of materials includes, for example, molybdenum disulfide (MoS_2_), tungsten disulfide (WS_2_) [11], hexagonal boron nitride (h-BN) [12,13,14], and graphite (C), etc. [15,16,17,18]. The factors influencing their application, besides price, can be resistance to high temperatures [7,19,20], reactivity, as well as the harmful impact on health [21]. To be used best, they must be placed in a tribological node.

Technical solutions for placing layered materials, e.g., boron nitride, in a friction node on several methods [7,10,22,23,24]. The material can be inserted when producing of the elements that work together, e.g., manufacturing products made of sintered powders. Technical solutions for placing layered materials, e.g., boron nitride, in a friction node are based on several methods [7,10,22,23,24]. The material can be inserted when producing the elements that work together, e.g., manufacturing products made of sintered powders. However, it is not the best solution from an economic point of view. It may lead to high consumption of layered materials, especially since they are subjected to high temperatures that accompany sintering during the sintering process. The matrix material may lose its properties as a result. More specialized methods, which involve targeted placement of the layered material, such as filling specially prepared “pockets” on the working surface, or using the sputtering laser technique, enable conserving these materials. However, the technology itself is not the most straightforward [7,20,21,25]. Due to the low difficulty level and the specific nature of the process, the simplest method is to introduce layered additives into the oil while being prepared for work [26]. However, due to the plate shape, difficulties may arise in obtaining stable mixtures (without clearly noticeable sedimentation) with base oils. Considering such particle’s ratio of surface area to volume or mass, one should expect that the smaller such a particle is, the better it should be able to form a stable mixture with a base oil. In this case, layered materials are not exposed to high temperatures and are better able to maintain their properties.

It is known that most lubrication systems are equipped with filters protecting against the flow of various pollutants in lubricant oils. Such filters stop larger particles of layered materials, which limits the usage of these materials. So just for this reason alone, it would be preferable to use layered materials with as small particles as possible. It can also be expected that such particles, similar in shape to spheres, would also better fill surface irregularities and spaces between elements working together in a tribological node [27]. At the same time, as reported by Gupta et al. [28], the use of layered materials of smaller-sized particles in base oils provides better anti-seizure properties [28,29]. A similar relation is observed when introducing layered materials directly during the preparation of friction subassemblies in the sintering process [10].

Hexagonal boron nitride h-BN was selected as the research material. It is not a material that provides the least friction. Still, compared to others, such as tungsten disulfide (WS_2_) or molybdenum disulfide (MoS_2_), it is safe for health, doesn’t react with other substances, and is relatively inexpensive and readily available. Furthermore, as an additive, it is unmatched among other materials in its resistance to high temperatures while retaining its structure and properties [7,20]. Most studies conducted to date focus on determining the effects of such additives whose particle sizes were relatively large; only the paper of M. K. Gupta et al. [28] addresses the need for a comparison of, among others, tribological properties of base oils with variously sized particles, containing 4% h-BN by weight [28]. During this time, our team researched an oil containing only h-BN with 70 nm particle size, using various concentrations of h-BN and various surfactant concentration (surface-active agent) levels. Everything indicates significant potential for nanomaterials to produce new, high-performance lubricant materials.

## 2. Materials and Methods

The pilot test program provided for preparing sample oils (PAO4, CB30), to which hexagonal boron nitride was introduced in specific amounts, then performing tests of physicochemical and tribological properties of such oils. The tests were intended to determine the impact of adding h-BN to base oil PAO4 on the properties in question; then, the results were compared with those obtained for the CB30 oil, which is designed for use in Diesel engines at low and medium loads. The purpose of the surfactant, which reduces surface tension in the oil (proprietary product), was to prevent any caking of h-BN particles and obtain a more stable h-BN suspension in the oil.

### 2.1. Test Equipment

Sample preparation and tests were conducted using equipment belonging to, among others, the research laboratory of SILESIA OIL SP. Z O.O. (SILESIA OIL SP. Z O.O. is name of company, Łaziska Górne, Poland).

Oil formulations were prepared using an IKA Plate, RCT Digital magnetic stirrer (IKA®-Werke GmbH & Co. KG, Staufen Germany), and IKA Ministar 20 Control mechanical stirrer (IKA®-Werke GmbH & Co. KG, Staufen Germany).

**Figure 2:**
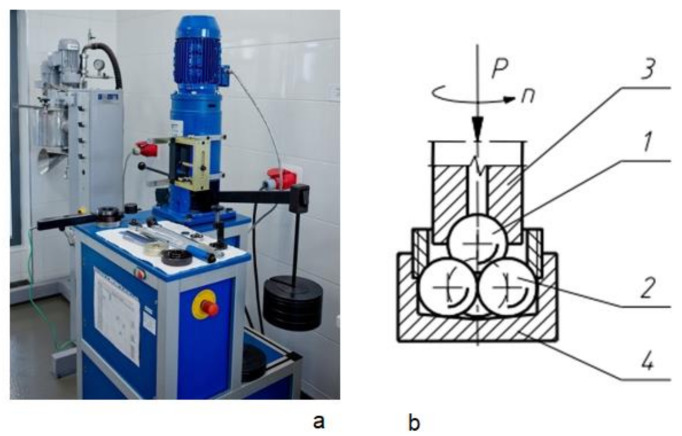
T02U I Silesia four-ball tester, (**a**) picture of the measuring device, (**b**) diagram of a four-ball friction node (1, top ball; 2, bottom balls; 3, 4, holders) [21].

Oil lubricity was tested using an ITEE T-02U universal four-ball tester (ITEE, Radom, Poland) designed for determining anti-seizure and anti-wear properties of lubricants and construction materials [30]. Friction torque, friction node load, and lubricant temperature were measured. The diagram, picture, and specification of a four-ball friction node are presented in Figure 2 and Table 1.

Wear spot size was measured using an OMO 06192/06193 optical microscope (Keyence International, Mechelen, Belgium), while images were taken using a PENTAX K-70 camera (Ricoh Polska Sp. z o.o., Warszawa, Poland).

Physicochemical tests were conducted using:

- a WTW pH-meter (Mettler Toledo, Columbus, USA) with a SenTix^®^41 pH electrode (Mettler Toledo, Columbus, USA), which was used to determine the pH values for oils;
- an Abbe refractometer (KERN Optics, Balingen, Germany), which was used to determine the critical angle of refraction when light rays pass from the air to the test oil;
- a Nima tensiometer (Nima Technology Ltd., Coventry, England), which was employed to determine surface tension using the tensiometric method;
- an apparatus comprising a Höppler viscosimeter (RHEOTEST, Medingen, Germany) and a thermostat was used for viscosity testing, and the test itself was conducted for temperatures of 40 and 95 °C;
- pycnometers (Archem, Kielce, Poland) placed in a thermostat (Merazet, Poznań, Poland) were used for oil density testing, and the test was conducted at temperatures of 20 °C.

### 2.2. Test Materials

Test samples were prepared using a base oil, surfactant, and boron nitride with 70 nm particle size (Hunan Fushel Technology Limited, Hunan, China).

#### 2.2.1. Hexagonal Boron Nitride

**Figure 3:**
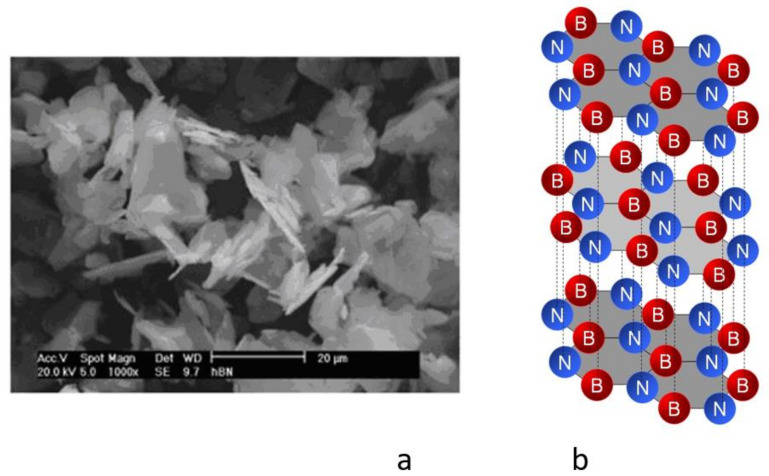
Hexagonal boron nitride (h-BN) (**a**) scanning microscope image; (**b**) crystallographic lattice diagram [7].

Hexagonal boron nitride is an inorganic chemical compound obtained by synthesis. It was first synthesized in 1842 by William H. Balmain [31]. In modern times it is obtained using high-energy methods producing boron-nitrogen bonds. It is found in three crystallographic forms (a, b, g). The a is characterized by a hexagonal structure, similar to graphite, designated h-BN—hexagonal boron nitride. It is a mild form of boron nitride with a plate structure and high anisotropy, used as a lubricant. It is chemically very stable used, among others, as an additive to cosmetics. The properties of hexagonal boron nitride are presented in Table 2. The scanning microscope image and crystallographic lattice diagram of hexagonal boron nitride (h-BN) are shown in Figure 3.

#### 2.2.2. Oils Used to Prepare the Test Samples

PAO4 oil (Exxon Mobil, Irving, USA) was used as the base oil for preparing the samples, while samples with the CB30 oil were used to compare the results obtained.

PAO4—and oil marketed under the name “Spectrasyn 4” by Brenntag Polska sp. z o.o., containing hydrogenated olefin oligomers obtained by catalytic polymerization of linear olefins, as well as a tetramer and a hydrated trimer. It is a polyalphaolefin oil with a viscosity class 4 [32].CB30 (ORLEN OIL Sp. z o.o., Kraków, Poland)—an oil designed for use in diesel engines at low and medium loads. Its main advantages are reduced wear of working parts, prevention of carbon and other deposits forming, prevention of piston ring jamming, dispersing and washing effect, high viscosity stability, oxidation resistance. It is a seasonal engine oil for trucks and machines with a viscosity class SAE 30 and a base number of 3.5 mg KOH/g.

The physicochemical properties of the oils (pH, refractive index, surface tension, aqueous extract reaction tests, dynamic and kinematic viscosity, and density) were tested at the Bioeletrochemistry Laboratory of the Faculty of Chemistry of the University of Bialystok. The results are summarized in Table 3 and Section 3.3.

#### 2.2.3. Anti-Caking Agent

Succinic acid imide (succinimide) (Merck, Darmstadt, Germany) was used to prevent hexagonal boron nitride particles caking and ensure longer retention of suspended particles in oil (slowing down sedimentation). The specification of a succinimide is presented in Table 4. The additive is a high-performance dispersing agent employed wherever there is a need to retain hydrocarbons or inorganic particles in an oil suspension. It has found use in the production of high-performance engine and transmission lubricants, and as an agent preventing oil deposit forming during downtimes, as a dispersing agent aiding in keeping process equipment clean in refineries, petrochemical industry, as well as gas and coke industry.

### 2.3. Sample Preparation for Testing

Test oil samples were prepared by measuring appropriate portions of oil, surfactant, and boron nitride, then mixing these substances with an electromagnetic and propeller stirrer. The mixing was done immediately before the tests, at ambient temperature, and for 5, 15, or 30 min for different samples, which contained either synthetic oil PAO4, which differed in the amount of the surfactant and boron nitride. In the case of the commercial oil (engine oil), CB30, no surfactant was used because it has a higher density, and it is easier to suspend nanoparticles in it. In these cases, only h-BN solids were added; five samples of the oil mixtures were prepared in this way. Additionally, tests were carried out for pure PAO4 and CB30 oils (without additives). The list of samples used in the research is presented in Table 5. Due to the large number of results obtained, the work presents only the data obtained for mixtures, the mixing time of which was 30 min.

### 2.4. Measuring Procedure

After obtaining a stable lubricant suspension by mixing, the sample was placed in the head of a four-ball tester, and a local wear spot test was performed (according to PN-C-04362: 2017-03). Mixtures obtained by mixing their constituents for 30 min were selected for testing at this stage, as this ensured producing a more stable suspension. The following testing conditions were adopted: ambient temperature 20 °C, rotation speed 1500 RPM, constant pressure 392 N, duration 3600 s. The balls used in the tests were made of chromium steel (100CR6). They had a diameter of 12.700 mm ± 0.0005 mm (characterizing data of the balls used in the tests: *Material*: AISA E-52100, *Hardness*: 64–66 HRC, *Surface Finish*: Grade 25 EP, *Company*: Stanhope-Seta).

The measurement was made in triplicate for each preparation, and the average steel ball wears spot diameter was calculated, which was then used to determine the limit wear load G_OZ_. Among the samples selected for the first test, some were further selected for a subsequent test to determine limit seizure load F_t_.

## 3. Results and Discussion

### 3.1. Wear Spot Measurement

**Figure 4:**
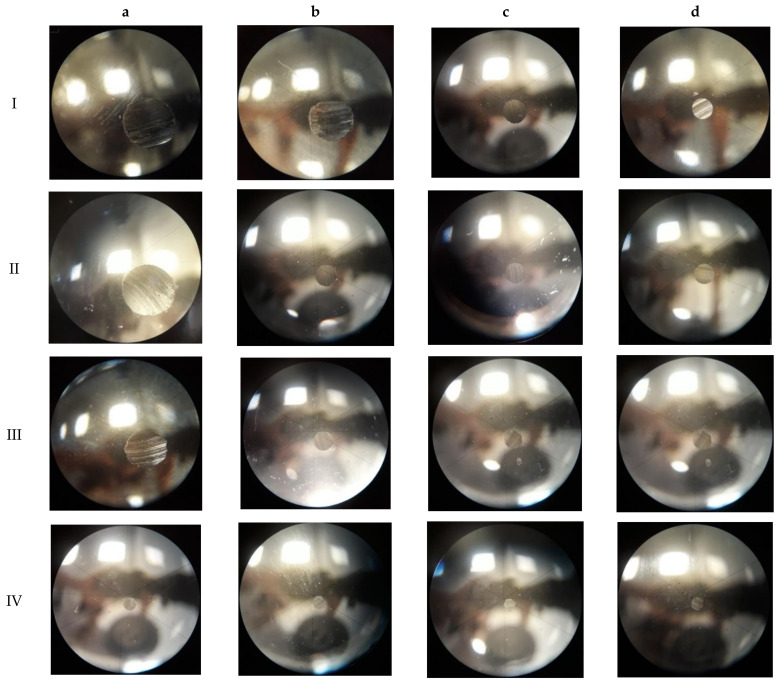
Pictures of sample wear spots following the test for samples are presented in Table 6.

**Figure 5:**
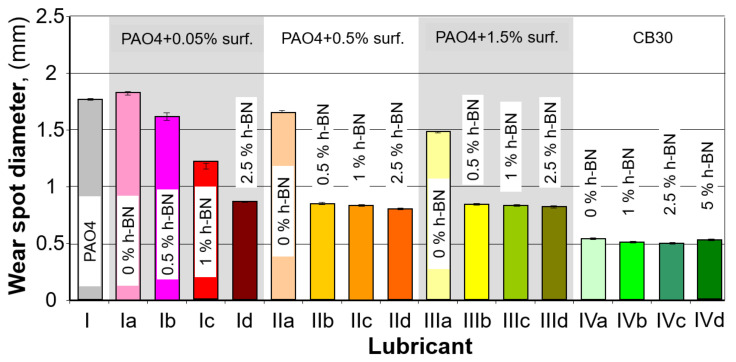
Summary of wear spot diameter measurement results.

The wear spot formed due to the tests performed was measured using a calibrated measurement microscope. The wear spots were measured along two perpendicular diameters, then images of the shape and type of the abrasion were taken. A summary of the pictures is shown below (Figure 4). The average wear spot diameter results are summarized in Table 6 and (Figure 5), where standard deviations for the measurements are also shown.

The average wear spot dimension results, shown above, were used to determine limit wear load G_OZ_ calculated using the formula [33].

(1) $$G_{\mathrm{OZ}}=0.52\frac{F}{d^{2}}[\frac{N}{mm^{2}}]$$

where:

F is the friction node load [N], F = 392 N;
D is the average wear spot diameter [mm];
0.52 is the factor accounting for the distribution of forces in the friction node.

**Figure 6:**
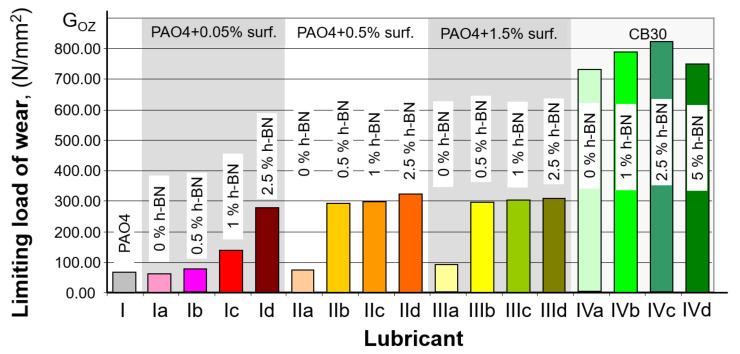
Limit wear load G_OZ_ as a function of base oil used and h-BN and surfactant addition.

Table 6 and Figure 6 show the calculated values of limit wear load G_OZ_.

The purpose of the surfactant (surfactant) is only to ensure better mixing of the h-BN with the oil and to create a stable suspension of the solid particles in the oil. Previous experiments show that h-BN particles without dispersing agent sediment more quickly, although the shorter the particle, the longer the falling time. An appropriate dispersing agent described in the literature was used [25]. The dispersing agent itself has little effect on the change of tribological properties. The abrasion diameter (flaw) size difference is within 5%. Using a dispersing agent in combination with hexagonal boron nitride allows for a positive effect.

Based on the obtained results of oil mixtures containing 0.05% of surfactant, it can be seen that the more h-BN particles are coated with the surfactant, the better the results of tribological properties are obtained. Too little surfactant may cause not all h-BN particles to be covered with it during the mixing process, which will cause some of them to fall off and not participate in the friction process. A clear improvement in tribological properties is already visible in the PA04 + 0.05% surf + 2.5% h-BN mixture. This means that the more h-BN particles there are, the more they participate in the friction process. This dependence is correct only up to a point—the content of 2.5% (higher contents of hexagonal boron nitride cause the sedimentation process). This regularity can be seen in further studies, during which increasing the amount of surfactant showed further improvement of the oil properties. The best result was obtained for the PA04 + 0.5% surf + 2.5% h-BN mixture. Further increasing the amount of surfactant did not bring about a significant improvement

As shown in Table 6 and the plot in Figure 5, the most significant changes in the size of the forming wear spot occurred when 0.5% wt. of the surfactant was added to the PAO4 base oil. In this case, the minor wear spot was obtained for the oil samples that, in addition to the 0.5% surfactant, also contained 2.5% wt. h-BN (273.70 [N/mm^2^]. As can be seen, the change is marked compared to the other samples in this group. For the other samples containing the PAO4 base oil, increasing the amount of surfactant to a small extent affected the size of the wear spot produced compared to the pure PAO4 case. As can be seen, 0.5% can be considered the optimum amount of surfactant; any further increase has a minor effect on the size of the wear spot produced. The results obtained also indicate a noticeable effect of h-BN (in the presence of a surfactant) on improving the results obtained; adding the substance to the PAO4 base oil enables obtaining about 50% smaller wear spot diameters; therefore, higher limit wear loads.

For the CB30 base oil, adding nano h-BN enables obtaining more minor wear spots and a limit seizure load higher by 13.6% for a 2.5% wt. h-BN content (compared to the oil without any layered material added). Further increasing the h-BN content to 5% led to achieving worse results. Therefore, using more significant amounts is not recommended, similar to particles with more significant grain sizes [20].

### 3.2. Seizure Load Determination

During the next test stage, some of the selected oil samples were subjected to tests on the four-ball tester to determine the seizure load. To this end, the work area of the four balls was filled with lubricant oil with suitable composition, while the load was continuously increased during work until a sudden increase in movement resistance, which was considered to be the moment that seizure began. The results thus obtained are shown in summary below, Table 7.

The results are shown below based on the results obtained and after calculating the possible standard deviation for the measurements.

Based on the results in Table 6 and Figure 6, it can be seen that in the case of PAO4 base oil, the best solution to obtain a higher seizure load limit is the use of a surfactant in an amount not exceeding 0.5% by weight and 2.5% h-BN additive. In this case, the limit value of the seizing load was about 28% higher than that obtained for the base oil.

It should also be noted that the addition of surfactant alone to the PAO4 base oil has no direct effect on the amount of the seizing load limit. Adding 2.5% h-HN to the base oil containing 0.05% of a surfactant allows obtaining a higher tripping load limit by about 8%. Increasing the amount of surfactant to 1.5% did not increase the ultimate seizure load, and the difference with 0.5% surfactant was within the measurement error.

The CB30 oil, in which the boron nitride nanoparticles in the amount of 2.5% was introduced, behaves similarly, which caused an increase of only less than 1% of the boundary welding load.

**Figure 7:**
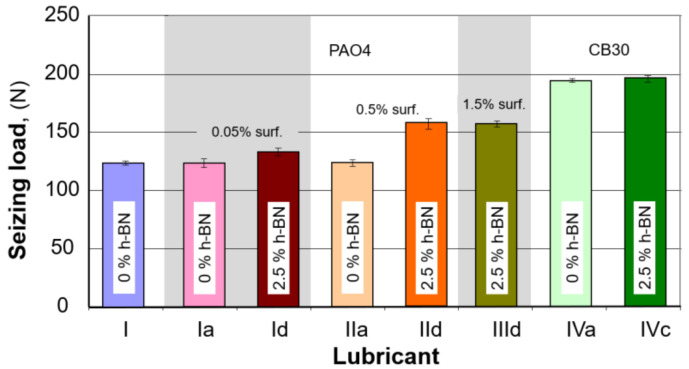
Seizure load values obtained.

The study’s primary purpose was to investigate how an h-BN nanopowder and a dispersing agent introduced in a lubricant oil affect its lubricating properties. The tests performed indicated that when the amount of h-BN particles and dispersing agent are adequately selected, it is possible to achieve better lubricating properties. The size of the wear spots formed due to the tests indicates that both hexagonal boron nitride nanoparticles and the dispersing agent used to affect the results achieved. As shown in Table 6 and Figure 6, h-BN content for base oil PAO4 should not exceed 2.5% wt, while it should not exceed 0.5% for the surfactant. An interesting case is the results obtained for lubricating oil containing 0.05 wt.%. surfactant and 2.5% nano h-BN (sample no. 1d, Figure 6). For the same amount of dispersant, the addition of more h-BN resulted in ~50% better results than with pure base oil; however, this was not accompanied by an increase in the value of the seizing load. Only increasing the amount of surfactant to 0.5% by weight made it possible to obtain a smaller scar diameter and a higher value of the seizing load. It can be seen here that the surfactant itself does not significantly affect the obtained results. Still, in combination with boron nitride, it allows to obtain a smaller scar diameter and, at the same time, a higher value of the seizing load. It is also notable that, as indicated by Figure 6 and Figure 7, the effect of the amount of h-BN is not unambiguous; everything means that the mixture preparation method and temperature in the lubricating socket are very important factors. The sample preparation stage focused on the mechanical process, which may not prepare the oil mixture sufficiently. All performed tests confirm that adding hexagonal boron nitride improves the lubricating properties of the resulting oil.

### 3.3. Physicochemical Properties

Table 8 presents the physicochemical results of selected mixtures of base oils with h-BN. We measured pH, surface tension, density, and dynamic and kinematic viscosity of various mixtures of PAO4 oil with the addition of a surfactant at a concentration of 0.5% and 1.5% and a solid substance—hexagonal boron nitride at concentrations of 0.5, 1.0, and 2.5%. In addition, we measured the physicochemical parameters for CB30 engine oil without surfactant and with hexagonal boron nitride at concentrations of 0.5, 1.0, and 2.5%.

The formation of stable boron nitride (h-BN) suspensions in oils is difficult due to the difference in density between the suspending phase (oil) and the suspended phase (h-BN), as a result of which there is a significant tendency to sedimentation. It is, therefore, necessary to stabilize the suspension. According to literature reports, detergents and dispersing additives stabilize boron nitride suspensions [7]. Thus, using the results of previous research carried out by many researchers [17,18,25], we used an appropriate surfactant when preparing a mixture of PAO4 oil with solid additives. The addition of a surfactant resulted in better dispersion of solid particles in the oil. In the analyzed samples, no sedimentation was observed in the case of nano-h-BN.

Viscosity has a decisive influence on the value of the internal friction resistance in the liquid and, above all, on the course of hydrodynamic lubrication. The oil’s high viscosity enables squeezing (displacing) from between the friction surfaces and thus prevents them from seizing. The higher the temperature and load of the tribological node, the higher the viscosity of the oil used [25]. The higher the viscosity, the thicker the lubricating film is formed on the friction surfaces, and the more difficult it is to force the oil out of the friction surfaces, which means that it better protects them from seizing. As shown from Table 8, the mixture containing 0.5% surfactant and 2.5% h-BN showed the highest viscosity for PAO4 oil.

Substances also exhibit the best lubricating properties that wet surfaces well, i.e., they have the lowest surface tension value. Their surface tension is significant for lubricating fluids, especially in micro and nonotribological systems. An important requirement for the lubricant is low surface tension because a low surface tension value corresponds to a low cohesion energy value. The weaker forces between the molecules make it easier to slide one after another. Thus, the lower the surface tension value, the lower the shear stresses and the less friction under boundary lubrication conditions. -for the liquid to lubricate a specific surface, it must cover (moisten) it. The liquid grease must moisten the surface so that the sliding does not occur on the surface’s non-greased part [25]. The lowest surface tension values were obtained in the PAO4 oil mixture with 0.5% surfactant and 2.5% h-BN.

Analyzing the results for the commercial oil (CB30; without the addition of surfactant), it can be concluded that, as in the case of PAO4 oil, the most optimal physical and sheath properties are demonstrated by oil mixtures with the addition of 2.5% h-BN: the lowest surface tension and the highest viscosity value.

Destructive methods (four-ball apparatus) are most often used to assess the durability of the boundary film of oils. The values of the seizing load are determined, i.e., the parameter characterizing the anti-seize properties of the lubricant. For the lubricating mixture to have good anti-seize properties, it must have the best physicochemical parameters: low surface tension and high viscosity value.

The obtained optimal physicochemical parameters of PAO4 oil mixtures with a surfactant at a concentration of 0.5% and the addition of 2.5% solids (70 nm h-BN) confirm the validity of the tests described based on measurements using a four-ball apparatus.

Analyzing the obtained results presented in Table 8 for synthetic and commercial oil, we can be noticed that the addition of 2.5% h-BN nanoparticles improves the physicochemical properties of the oils as indicated by the low surface tension value and high viscosity value.

## 4. Conclusions

Comparing the results of the research on the influence of h-BN nanoparticles on the tribological properties of base oil (PAO4) and commercial oil (CB30), it can be clearly stated that it has a positive effect in both cases, especially when it comes to the G_oz_ limit wear. At the same time, it should be noted that in the case of commercial oil, the seizing load was higher than in the case of the base oil, but no influence of h-BN on its growth was noticed.

Considering the obtained results, it can be concluded that adding the h-BN layered nanomaterial to the lubricating oil can be a good solution for getting a lubricating oil with ~27% greater galling load than to the base oil. It is possible provided that certain conditions for the preparation of the lubricating oil are met. In the case of PAO4 base oil, succinimide in an amount not exceeding 0.5% by weight should be used as a dispersing agent—hexagonal boron nitride (70 nm at 2.5 wt%). The obtained mixture should be stirred with a stirrer until a stable suspension of boron nitride in the oil is received for not less than 30 min. During long-term storage of the lubricating oil thus produced, sedimentation of h-BN particles is noticeable.

Analyzing the commercial oil CB30 and PAO4 oil results, oil mixtures demonstrate the most optimal physical and sheath properties with the addition of 2.5% h-BN: the lowest surface tension and the highest viscosity value.

All the tests were carried out to confirm that the introduction of hexagonal boron nitride improves the lubricating properties of the prepared oil. However, everything indicates that the appropriate amounts of a surfactant or hexagonal boron nitride play an important role in the friction processes and the results achieved, the method of preparing the oil mixture, or the thermal properties of h-BN. During the preparation of the oil mixtures, great difficulties were observed in keeping the h-BN suspension in the oil longer.

At the same time, the conducted research presented in work [10] and the present one confirm the validity of the previously proposed lamellar model and, at the same time, indicate further directions of research.

To investigate the processes taking place in more detail, it would be advisable to investigate further the influence of hexagonal boron nitride on the temperature of the lubricating nipple and the effect of the lubricating component mixing technique on the lubricating properties of the oil.

## Figures and Tables

**Table 1:** T02U tester specifications.

Parameter	Character/Value
Movement Type	Sliding or rolling
Contact Geometry	Point
Friction Node	Four ½″ balls
Test Material	Lubricants and construction materials
Friction Node Temperature	Stabilized from ambient temperature to 75 (°C) ± 2 °C, possible temperature range up to 175 (°C)
Spindle Rotation Speed	Seamless adjustable from 300 to 1800 (RPM)
Contact Load	Adjustable from 0 to 7850 (N) using a lever with a weight
Measured Parameter	Friction node temperature, load, movement resistance, rotation speed, vibration amplitude, time
Power Consumption	~2 (kW)
Power Supply	230 [V] 50 (Hz)

**Table 2:** Hexagonal boron nitride.

Properties of Hexagonal Boron Nitride	
Molecular Weight (g/mol)	24.82
Metallic Properties	non-metal
Appearance	white
Mohs scale of Hardness	1.5 ÷ 2
Density, (g/cm^3^)	1.7 ÷ 2.2
Melting Point, (°C)	1185
Lubrication Temperature Range, (°C)	−40 ÷ 870

**Table 3:** Test oil specifications.

Physicochemical Properties	PAO4	CB30
Flash Point, min (°C)	204	210
Pour Point, max (°C)	−57	−24
pH	6.86	6.32
Aqueous Reaction pH	6.92	6.52
Refractive Index	1.4553	1.4765
Acid Number, (mg KOH/g of oil)	0.01	3.5
Dynamic Viscosity at 40 °C (mPa·s)	12.58	44.37
Dynamic Viscosity at 95 °C (mPa·s)	3.26	8.33
Kinematic Viscosity at 40 °C, (mm^2^/s)	16.39	51.48
Kinematic Viscosity at 95 °C, (mm^2^/s)	3.94	9.66
Surface Tension (mN/m)	45.20	44.70
Viscosity Index	105	95
Density at 20 °C (g/cm^3^)	0.8282	0.8619

**Table 4:** Succinimide specifications.

Physicochemical Properties	Succinic Acid Imide
Flashpoint, min (°C)	190
Acid Number, (mg KOH/g of oil)	42
Kinematic Viscosity at 100 °C, (mm^2^/s)	440
Kinematic Viscosity at 40 °C, (mm^2^/s)	105
Pour Point, max (°C)	−24
Density (g/cm^3^)	0.927
Appearance	Dark brown, viscous liquid

**Table 5:** Summary of test oil mixture compositions.

Oil and % wt of Surfactant	h-BN Content (% wt)			
	0.0	0.5	1.0	2.5
**PA04**, 0%	I	**Not included**	**Not included**	**Not included**
**PA04**, 0.05%	Ia	Ib	Ic	Id
**PA04**, 0.5%	IIa	IIb	IIc	IId
**PA04**, 1.5%	IIIa	IIIb	IIIc	IIId
**Oil and % wt of surfactant**	**0.0**	**1.0**	**2.5**	**5**
**CB30**, 0%	IVa	IVb	IVc	IVd

**Table 6:** Average wear spot diameter.

Sample No.	Composition	Average Wear Spot Diameter (mm)				Standard Deviation (S_x_)	Limit Wear Load (N/mm^2^)
		Measurement			Average		
		1	2	3			
I	PAO 4	1.76	1.76	1.77	1.76	0.01	65.58
Ia	PAO 4 + 0.05% surf	1.80	1.93	1.85	1.86	0.07	58.92
Ib	PAO 4 + 0.05% surf + 0.5% h-BN	1.60	1.70	1.60	1.63	0.05	76.41
Ic	PAO 4 + 0.05% surf + 1.0% h-BN	1.18	1.20	1.26	1.21	0.04	138.46
Id	PAO 4 + 0.05% surf + 2.5% h-BN	0.87	0.86	0.86	0.86	0.01	273.48
IIa	PAO 4 + 0.5% surf	1.64	1.74	1.66	1.68	0.05	72.22
IIb	PAO 4 + 0.5% surf + 0.5% h-BN	0.86	0.84	0.83	0.84	0.02	286.84
IIc	PAO 4 + 0.5% surf + 1.0% h-BN	0.84	0.84	0.82	0.83	0.01	293.53
IId	PAO 4 + 0.5% surf + 2.5% h-BN	0.80	0.79	0.81	0.80	0.01	318.50
IIIa	PAO 4 + 1.5% surf	1.50	1.55	1.51	1.52	0.03	88.23
IIIb	PAO 4 + 1.5% surf + 0.5% h-BN	0.84	0.84	0.83	0.84	0.01	291.20
IIIc	PAO 4 + 1.5% surf + 1.0% h-BN	0.82	0.84	0.82	0.83	0.01	298.28
IIId	PAO 4 + 1.5% surf + 2.5% h-BN	0.83	0.83	0.8	0.82	0.02	303.15
IVa	CB 30	0.53	0.53	0.54	0.53	0.01	716.63
IVb	CB 30 + 1.0% h-BN	0.50	0.52	0.51	0.51	0.01	783.70
IVc	CB 30 + 2.5% h-BN	0.50	0.51	0.49	0.50	0.01	815.36
IVd	CB 30 + 5.0% h-BN	0.51	0.53	0.53	0.52	0.01	744.27

**Table 7:** Seizure load F_t_ results summary.

Sample No.	Composition	Weld Point (daN)				Standard Deviation (S_x_)
		Measurement				
		1	2	3	Average	
I	PAO4	12,3.43	12,3.65	12,3.60	12,3.56	0.12
Ia	PAO 4 + 0.05% surf	12,3.86	12,3.38	12,3.41	12,3.55	0.27
Id	PAO 4 + 0.05% surf + 2.5% h-BN	13,3.79	13,3.90	13,4.25	13,3.98	0.24
IIa	PAO 4 + 0.5% surf	12,3.56	12,3.37	12,3.75	12,3.56	0.19
IId	PAO 4 + 0.5% surf + 2.5% h-BN	15,8.39	15,8.40	15,7.84	15,8.21	0.32
IIId	PAO 4 + 1.5% surf + 2.5% h-BN	15,7.97	15,8.32	15,8.25	15,8.18	0.19
IVa	CB 30	19,4.23	19,4.37	19,4.15	19,4.25	0.11
IVc	CB 30 + 2.5% h-BN	19,6.40	19,6.17	19,5.17	19,6.19	0.20

**Table 8:** Physicochemical results summary.

Sample No.	Composition	Measured Parameters							
		pH	Refractive Index	Surface Tension (mN/m)	Dynamic Viscosity (mPa·s)		Kinematic Viscosity (mm^2^/s)		Density at 20 °C (g/cm^3^)
					At 40 °C	At 95 °C	At 40 °C	At 95 °C	
I	PAO4	6.86	1,4553	45.2	12.58	3.26	16.39	3.94	0,8282
IIa	PAO 4 + 0.5% surf	7.08	1,4550	43.6	13.08	3.35	17.22	4.69	0,8293
IIb	PAO 4 + 0.5% surf + 0.5% h-BN	-	1,4553	45.1	13.80	3.41	17.02	4.34	0,8299
IIc	PAO 4 + 0.5% surf + 1.0% h-BN	-	1,4556	43.2	14.45	3.49	16.67	4.01	0,8310
IId	PAO 4 + 0.5% surf + 2.5% h-BN	-	1,4556	40.0	15.50	3.90	17.07	5.23	0,8322
IIIa	PAO 4 + 1.5% surf	7.65	1,4555	41.2	14.03	3.72	16.23	4.89	0,8333
IIIb	PAO 4 + 1.5% surf + 0.5% h-BN	-	1,4560	50.7	14.66	3.78	16.22	4.88	0,8345
IIIc	PAO 4 + 1.5% surf + 1.0% h-BN	-	1,4562	53.1	14.79	3.81	15.81	4.68	0,8364
IIId	PAO 4 + 1.5% surf + 2.5% h-BN	-	1,4562	51.7	15.08	3.88	14.70	4.57	0,8376
Iva	CB30	6.32	1,4765	44.7	44.37	8.33	51.48	9.66	0,8619
IVb	CB 30 + 0.5% h-BN	-	1,4774	48.5	47.40	10.26	42.38	9.18	0,8655
IVc	CB 30 + 1.0% h-BN	-	1,4774	44.0	51.67	11.80	46.37	9.55	0,8780
IVd	CB 30 + 2.5% h-BN	-	1,4777	42.8	52.99	12.05	52.61	10.65	0,8889

## Data Availability

All the data is available within the manuscript.
